# NOD1/RIP2 signalling enhances the microglia-driven inflammatory response and undergoes crosstalk with inflammatory cytokines to exacerbate brain damage following intracerebral haemorrhage in mice

**DOI:** 10.1186/s12974-020-02015-9

**Published:** 2020-12-01

**Authors:** Miao Wang, Xinchun Ye, Jinxia Hu, Qiuchen Zhao, Bingchen Lv, Weijing Ma, Weiwei Wang, Hanhan Yin, Qi Hao, Chao Zhou, Tao Zhang, Weifeng Wu, Yan Wang, Mingyue Zhou, Cong-hui Zhang, Guiyun Cui

**Affiliations:** 1 Department of Neurology, Xuzhou first People’s Hospital, The Affiliated Xuzhou Municipal Hospital of Xuzhou Medical University, Xuzhou Medical University, No. 269 University Road, Tongshan District, Xuzhou, Jiangsu China; 2 Institute of Nervous System Diseases and Department of Neurology, The Affiliated Hospital of Xuzhou Medical University, Xuzhou Medical University, No. 99 West Huaihai Road, Xuzhou, 221006 Jiangsu Province China; 3 Department of Neurology, Mass General Institute of Neurodegenerative Diseases, Massachusetts General Hospital and Harvard Medical School, Charlestown, USA; 4 Department of Rehabilitation Medicine, Linyi Cancer Hospital, Linyi, Shandong China; 5 Department of Neurology, Second Affiliated Hospital of Xuzhou Medical University, Xuzhou, Jiangsu China

**Keywords:** NOD1, RIP2, Microglial activation, Inflammatory response, Intracerebral haemorrhage

## Abstract

**Background:**

Secondary brain damage caused by the innate immune response and subsequent proinflammatory factor production is a major factor contributing to the high mortality of intracerebral haemorrhage (ICH). Nucleotide-binding oligomerization domain 1 (NOD1)/receptor-interacting protein 2 (RIP2) signalling has been reported to participate in the innate immune response and inflammatory response. Therefore, we investigated the role of NOD1/RIP2 signalling in mice with collagenase-induced ICH and in cultured primary microglia challenged with hemin.

**Methods:**

Adult male C57BL/6 mice were subjected to collagenase for induction of ICH model in vivo. Cultured primary microglia and BV2 microglial cells (microglial cell line) challenged with hemin aimed to simulate the ICH model in vitro. We first defined the expression of NOD1 and RIP2 in vivo and in vitro using an ICH model by western blotting. The effect of NOD1/RIP2 signalling on ICH-induced brain injury volume, neurological deficits, brain oedema, and microglial activation were assessed following intraventricular injection of either ML130 (a NOD1 inhibitor) or GSK583 (a RIP2 inhibitor). In addition, levels of JNK/P38 MAPK, IκBα, and inflammatory factors, including tumour necrosis factor-α (TNF-α), interleukin (IL)-1β, and inducible nitric oxide synthase (iNOS) expression, were analysed in ICH-challenged brain and hemin-exposed cultured primary microglia by western blotting. Finally, we investigated whether the inflammatory factors could undergo crosstalk with NOD1 and RIP2.

**Results:**

The levels of NOD1 and its adaptor RIP2 were significantly elevated in the brains of mice in response to ICH and in cultured primary microglia, BV2 cells challenged with hemin. Administration of either a NOD1 or RIP2 inhibitor in mice with ICH prevented microglial activation and neuroinflammation, followed by alleviation of ICH-induced brain damage. Interestingly, the inflammatory factors interleukin (IL)-1β and tumour necrosis factor-α (TNF-α), which were enhanced by NOD1/RIP2 signalling, were found to contribute to the NOD1 and RIP2 upregulation in our study.

**Conclusion:**

NOD1/RIP2 signalling played an important role in the regulation of the inflammatory response during ICH. In addition, a vicious feedback cycle was observed between NOD1/RIP2 and IL-1β/TNF-α, which could to some extent result in sustained brain damage during ICH. Hence, our study highlights NOD1/RIP2 signalling as a potential therapeutic target to protect the brain against secondary brain damage during ICH.

**Supplementary Information:**

The online version contains supplementary material available at 10.1186/s12974-020-02015-9.

## Introduction

Given the high morbidity and mortality of intracerebral haemorrhage (ICH) [1], concerns about identifying an effective treatment for ICH have been increasing. However, only identification of the mechanism underlying ICH-induced damage can address this concern. Accumulating evidence has demonstrated that the second wave of innate immune-induced inflammatory injury, rather than direct damage caused by hematoma-mediated compression, is the main contributor to a series of damaging events following ICH [2–4], including brain oedema, BBB damage, cell death, severe neurological dysfunctions, and even death. Secondary neuroinflammation is characterized by an innate immune response and subsequent inflammatory cytokine production. The innate immune response mainly involves the activation of microglia and the infiltration of other blood-derived immune cells [5, 6]. Microglia act as a major innate immune cell in the central nervous system (CNS) and can be induced to transition from the resting form to the activated form after sensing brain tissue injury [7, 8]. Activated microglia are responsible for the production of inflammatory cytokines, such as IL-1β, TNF-α, and IL-6 [9–12]. Therefore, targeting the innate immune response has become a major goal of ICH treatment.

Nucleotide-binding oligomerization domain 1 (NOD1), a member of the NOD-like receptor (NLR) family, has been found to be an intracytoplasmic innate immune receptor that can drive the innate immune response and trigger a cascade of inflammatory signalling events upon sensing pathogens or tissue injury [13–15]. Evidence has shown that NOD1 is partially responsible for the pathogenesis of polymorphonuclear leukocyte (PMN)-induced liver ischemia injury, and deletion of NOD1 has a neuroprotective effect on PMN-dependent liver disease [16, 17]. Proinflammatory macrophage activation and neutrophil infiltration in adipose tissue during consumption of a high-fat diet could be prevented upon NOD1 depletion [18]. In addition, it has been reported that NOD1 and its downstream molecular RIP2 [19, 20] play a pivotal role in modulating nuclear factor kappa B (NF-κB) and mitogen-activated protein kinase (MAPK) signalling activation [21, 22], which is associated with neuroinflammation [23–25]. These findings suggest that NOD1/RIP2 signalling plays an important role in the inflammatory response. Thus, we asked whether NOD1/RIP2 signalling participates in the pathogenesis of ICH, and more specifically, whether NOD1/RIP2 signalling mediates microglial activation and subsequent proinflammatory signalling during ICH.

In the present study, we explored the role of NOD1/RIP2 signalling in the regulation of the inflammatory response during ICH. We found that NOD1 and RIP2 were increased in response to ICH and contributed to microglial activation and the inflammatory response. However, surprisingly, we identified a crosstalk between NOD1/RIP2 and the inflammatory cytokines IL-1β and TNF-α. NOD1 and RIP2 activation not only had positive impacts on inflammatory cytokines, but were also affected by them. The formation of this vicious cycle may illuminate the context of severe inflammation during ICH. Therefore, blockade of NOD1/RIP2 signalling would be a promising therapeutic approach for ICH.

## Materials and methods

### Mice

Male C57BL/6 mice at the age of 2–3 months were used in all animal experiments, and all mouse procedures were approved by the institutional Animal Use and Care Committee of XuZhou Medical University. Mice were fed in a strict pathogen-free environment with a 12-h light/dark cycle and free access to water.

### Induction of the ICH model in mice

Induction of the ICH model was based on a previous report [26]. In brief, mice were first anesthetized with pentobarbital sodium (50 mg/kg) and then placed on a stereotaxic frame. A cranial burr hole was drilled with a well velocity meter 2.5 mm lateral to the midline. Collagenase VII (0.045 U in 1 μl of sterile saline) (Sigma-Aldrich; Germany) was injected at a rate of 0.2 μl/min into the left striatum, which was located 0.2 mm anterior, 2.5 mm lateral, and 3.5 mm deep relative to the bregma [27]. After injection, the needle was left for 5 min to avoid the reflux. The sham group underwent all procedures except collagenase injection.

### Cell culture and in vitro ICH model

#### Primary microglial culture

New-born mice at days 1–3 were used for isolation of the primary microglia. Cortex-striatum tissues were extracted from the brain and cut into tiny pieces, followed by digestion with trypsin for 15 min, when it was possible to adequately dissociate the tissues into mixed glia. These glia were then plated into T-25 culture flasks containing DMEM/F12 with 10% foetal bovine serum, GlutaMAX (Invitrogen), and 1% penicillin/streptomycin. After culture in a 5% CO2/37 °C incubator for 14 days, the flasks were shaken at 220 rpm for 4 h at 37 °C to harvest the primary microglia. Thereafter, the microglia were plated in 6-well plates at a density of 5 × 10^5^ cells per well for subsequent experiments.

#### Microglial cell line culture

The microglial cell line BV2 was incubated in 6-well plates containing Dulbecco’s modified Eagle’s medium (DMEM)/high glucose supplemented with 10% foetal bovine serum, 120 U/ml penicillin, and 100 mg/l streptomycin in an incubator containing 5% CO_2_ at 37 °C.

#### Induction of an in vitro ICH model

Based on a report that hemin plays an important role in the pathology of ICH [28] and the use of hemin to induce ICH in an in vitro study [29], hemin (Sigma-Aldrich, Germany) was used to stimulate cultured primary microglia and the BV2 microglial cell line in our in vitro ICH model.

### Drug administration

(1) The following inhibitors used in our study were purchased from Selleck Chemicals (Houston, TX, USA): (i) ML130, a highly selective and effective inhibitor of NOD1 (20 μM in vivo and in vitro); (ii) GSK583, a specific RIP2 inhibitor (20 μM in vivo and in vitro); (iii) BAY11-7082, an NF-κB signalling inhibitor capable of inhibiting IκBα degradation (10 μM in vivo and in vitro); (iv) SP600125, a JNK kinase inhibitor (10 μM in vivo and in vitro); and (v) SB202190, a P38 kinase inhibitor (10 μM in vivo and in vitro). These inhibitors were dissolved in dimethyl sulfoxide (DMSO) and injected into mice via the lateral ventricle (positioned 1.5 mm posterior, 1.0 mm lateral, and 3.2 mm deep relative to the bregma) [30] 30 min prior to ICH induction in mice. For the in vitro intervention, microglia were pretreated with these inhibitors for 1 h followed by hemin induction.

(2) The selective NOD1 agonist C12-iE-DAP (Sigma-Aldrich, Merck), diluted in purified water, was injected into mice via the lateral ventricle at 10 min following ICH induction and administered to cultured microglia at 10 min post-hemin exposure.

(3) Recombinant mouse IL-1β/TNF-α (Minneapolis, MN), diluted in sterile PBS, was injected into mice via the local right striatum or administered to cultured primary microglia for 24 h.

(4) In all experiments, the mice in the sham group or the microglia in the control group received an equal volume of solvent (DMSO or purified water, or sterile PBS) used for drug dissolution.

### Behaviour assessment

The modified Neurological Severity Score (mNSS) [31], which includes motor, sensory, balance, and reflex tests, together with the corner-turning test, was employed to evaluate neurological deficits before ICH and at 3 days post ICH. The score ranges from 0 to 18 points, and 1 point is given for failing to perform one task. Mice with more severe damage will receive higher scores. The corner-turning test was performed according to a previous study [32]. The turn made by the mouse was recorded as either right or left, and then, the percentage of left turns was calculated.

### Brain water content

The brains obtained from mice under deep anaesthesia were divided into three parts, namely, the ipsilateral and contralateral hemispheres and the cerebellum, and the parts were then weighed to determine the wet weight, placed into the oven at 100 °C for 24 h, and reweighed to determine the dry weight. The brain water content was calculated according to the following formula: (wet weight-dry weight)/wet weight*100% [33].

### Nissl and immunofluorescence staining

After sequential perfusion of the mice with PBS and 4% paraformaldehyde via the heart, the brains were removed and fixed in 4% paraformaldehyde overnight. Thereafter, brain specimens were sectioned coronally into 40-50-μm-thick sections from the frontal lobe to the visual cortex, followed by Nissl or immunofluorescence staining.

1. The major steps of Nissl staining were conducted as follows: the sections were incubated in 100, 95, and 80% ethanol for 30 s and then treated with FD Cresyl Violet Solution^TM^ (FD Neoro Technologies, Columbia, MD, USA) for 2 min, followed by dehydration through a series of alcohol concentrations and sealing with neutral resin. The injury volumes of the stained sections were measured with ImageJ software and calculated via indirect methods as previously reported [34].
2. For immunofluorescence staining, the sections were preblocked and permeabilized with a mixture containing 90% PBS, 10% donkey serum, and 0.5% Triton X-100 for 1 h and then coincubated with rabbit anti-Iba-1 (1:500, WAKO, Japan) and rat anti-CD68 (1:500, Abcam, Cambridge, UK) antibodies overnight at 4 °C. Then, these sections were washed 3 times with PBS, followed by incubation with an Alexa Fluor 555 or Alexa Fluor 488 secondary antibody (1:500, Protein tech Group, Inc, Chicago, IL, USA) at 37 °C for 2 h. Finally, the positive cells from three different areas of tissue surrounding the hematoma in each brain section were analysed with ImageJ software.

### Immunohistochemistry staining

After transcardially perfusing mice with 4% paraformaldehyde, the brains were removed and fixed in the same fixative overnight. They were then paraffin-embedded and sectioned coronally into 4 μm. This process was followed by deparaffinization with xylene and graded ethanols sequentially. Next, 10 mmol citrate buffer was conducted for antigen retrieval, 3% hydrogen peroxide was applied for endogenous peroxidase activity blockade, and 5% bovine serum albumin was performed for blocking the nonspecific staining. Thereafter, these sections were incubated with rabbit anti-Iba-1 (1:500, WAKO, Japan) at 4 °C overnight. The sections were then incubated with a secondary antibody for 1.5 h at room temperature, followed by DAB staining for 3 min. Finally, immunopositive cells from three different areas around the hematoma were analyzed by three observers blind to the mice group information.

### Western blot analysis

The main protein extraction and western blot procedures were conducted according to previously described methods [34]. Perihematomal tissues and hemin-induced microglia were collected. These tissues were lysed with RIPA buffer for 10 min and centrifuged at 4 °C at 13,000*g* for 30 min, and the protein in the supernatant was collected for western blot analysis. Equal amounts of protein were separated by electrophoresis and transferred to nitrocellulose membranes. The membranes were first blocked with 5% non-fat milk and then incubated with the following primary antibodies overnight at 4 °C: rabbit anti-NOD1 (1:1000), rabbit anti-RIP2 (1:1000), rabbit anti-IL-1β (1:1000), rabbit anti-TNF-α (1:1000), rabbit anti-iNOS (1:1000), rabbit anti-total P38 (1:2000), rabbit anti-p-P38 (1:2000), rabbit anti-total JNK (1:5000), rabbit anti-p-JNK (1:5000), and rabbit anti-IκBα (1:5000). All of these antibodies were obtained from the company of Cell Signalling Technology.

### Experimental protocol

**Fig. 1:**
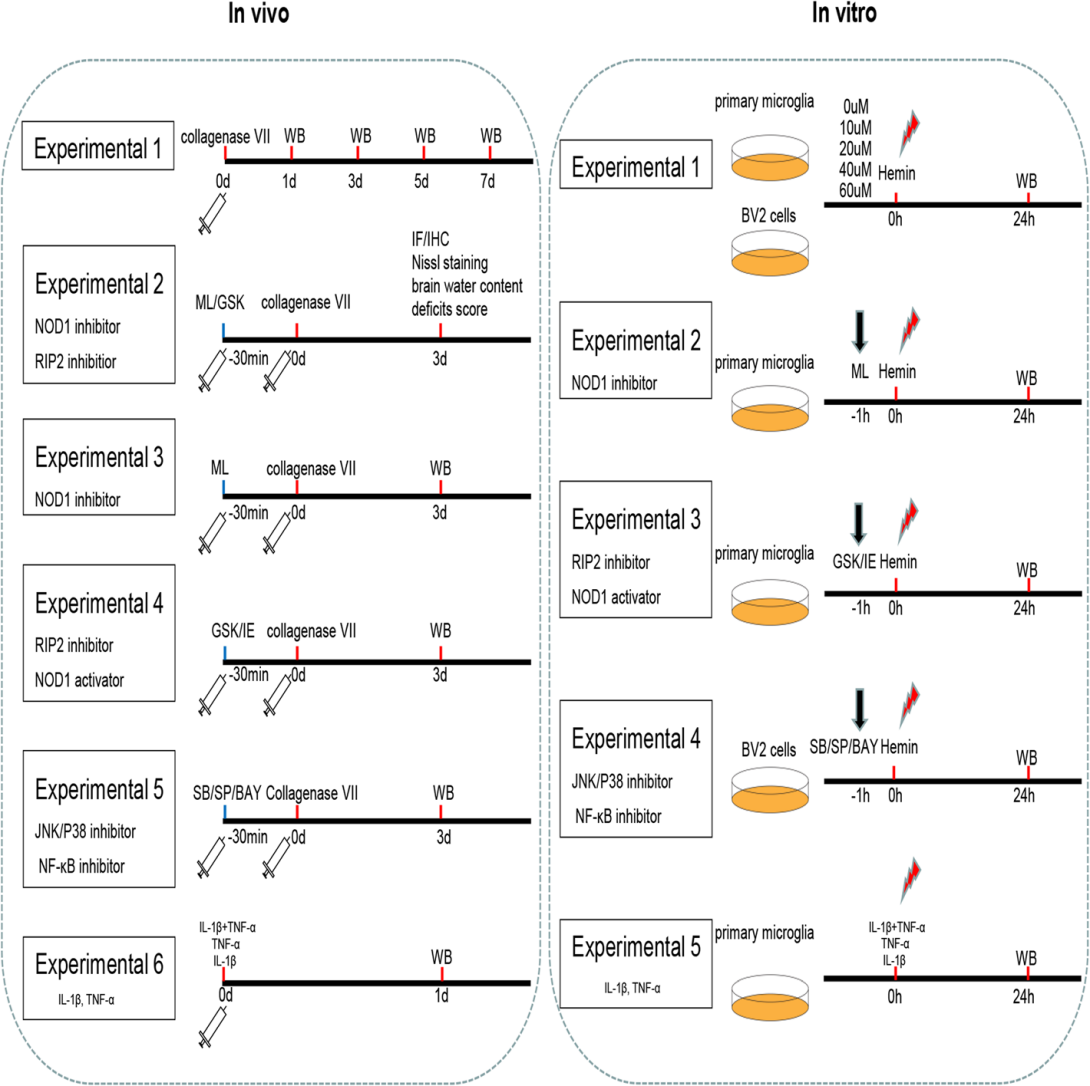
Experimental design of the in vivo and vitro study. ICH, intracerebral hemorrhage; WB, western blot; IF, immunofluorescence; IHC, immunohistochemistry; ML, ML130; GSK, GSK583; iE, C12-iE-DAP; SP, SP600125; SB, SB202190; BAY, BAY11-7082; d, day; h, hour

This study was mainly divided into two parts: in vivo and in vitro. The design of the experiment is shown in Fig. 1.

Part 1 In vivo study

All mice were randomized into the following five experiments:

1. To determine NOD1 and RIP2 expression at different time points post ICH, the mice were randomly divided into five groups: sham, ICH-1d, ICH-3d, ICH-5d, and ICH-7d (*n* = 3/group).
2. To detect the effects of NOD1 inhibition and RIP2 inhibition on ICH-induced brain injury, the ICH + DMSO, ICH + ML130(ML), and ICH + GSK583(GSK) groups were used to evaluate neurological deficits (*n* = 10/group), assess brain water content (*n* = 6/group), and test injury volume (*n* = 6/group). The sham, ICH + DMSO, ICH + ML130(ML), and ICH + GSK583(GSK) groups were used to assess microglial activation (*n* = 6/group).
3. To explore the effects of NOD1 inhibition on the inflammatory response, the mice were randomized into four groups: sham, ICH, ICH + DMSO, and ICH + ML130 (ML) (*n* = 3/group).
4. To explore the effects of RIP2 inhibition on the inflammatory response, the mice were randomized into five groups: sham, ICH + DMSO, ICH + iE-DAP (iE), ICH + GSK583 (GSK), and ICH+iE-DAP (iE) + GSK583 (GSK) (*n* = 3/group).
5. To investigate whether the NOD1/RIP2-induced inflammatory factors were dependent on JNK/P38 MAPK and NF-κB signalling, the mice were divided into five groups: sham, ICH + DMSO, ICH + SP600125 (SP), ICH + SB202190 (SB), and ICH + BAY11-7082 (BAY) (*n* = 3/group).
6. To explore whether NOD1 and RIP2 expression could be regulated by IL-1β and TNF-α, the mice were divided into four groups: sham, IL-1β, TNF-α, and IL-1β + TNF-α (*n* = 3/group).

Part 2 In vitro study

1. To determine NOD1 and RIP2 expression in cultured primary microglia and BV2 cells after exposure to different doses of hemin, the microglia were randomly divided into five groups: control, Hemin-10 μM, Hemin-20 μM, Hemin-40 μM, and Hemin-60 μM (*n* = 3/group).
2. To explore the effects of NOD1 inhibition on inflammatory cytokine production and JNK/P38 MAPK and NF-κB signalling pathways, primary microglia were randomized into four groups: control, Control + ML130 (ML), Hemin, and Hemin + ML130 (ML) (*n* = 3/group).
3. To explore the effects of RIP2 inhibition on inflammatory cytokine production and JNK/P38 MAPK and NF-κB signalling pathways, primary microglia were randomized into five groups: control, Hemin + DMSO, Hemin + iE-DAP (iE), Hemin + GSK583 (GSK), and Hemin + iE-DAP (iE) + GSK583 (GSK) (*n* = 3/group).
4. To investigate whether NOD1/RIP2-induced inflammatory cytokine production was dependent on JNK/P38 MAPK and NF-κB signalling, BV2 cells were divided into five groups: control, Hemin + DMSO, Hemin + SP600125 (SP), Hemin + SB202190 (SB), and Hemin + BAY11-7082 (BAY) (*n* = 3/group).
5. To explore whether NOD1 and RIP2 expression could be regulated by IL-1β and TNF-α, the cultured primary microglia were divided into four groups: control, IL-1β, TNF-α, and IL-1β + TNF-α (*n* = 3/group).

### Statistical analysis

GraphPad Prism 8 was employed for statistical analysis of the data in this study. All data are presented as the mean ± SEM. One-way ANOVA with Tukey’s post hoc test was applied for the comparison among multiple groups. Student’s *t* test was used to compare differences between two groups. *P* < 0.01 indicated a significant difference.

## Results

### NOD1 and RIP2 expression displayed dramatic increases following ICH

**Fig. 2:**
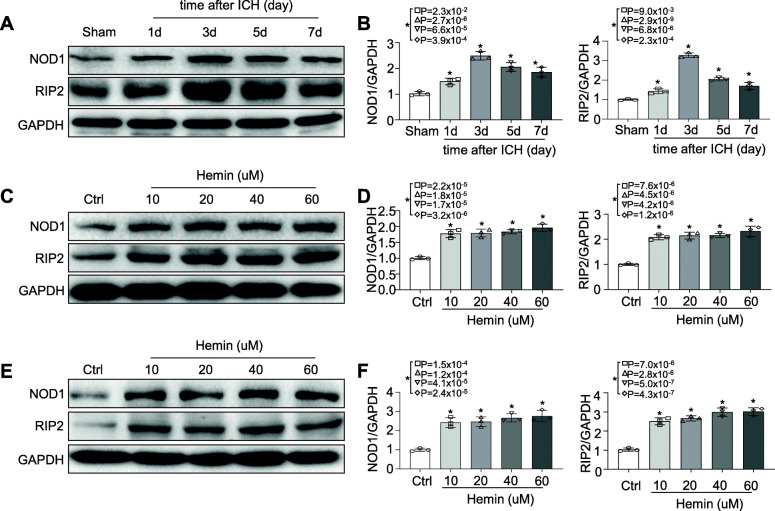
NOD1 and RIP2 expression were upregulated following ICH. Western blot of NOD1 and RIP2 **a**, **b** in the brains of mice on the indicated days following ICH (*n* = 3 mice for each group; *P < 0.01 vs. the sham group), **c**, **d** in primary microglia, and **e**, **f** in BV2 cells challenged with hemin at the indicated doses for 24 h (*n* = 3 experiments for each group; *P < 0.01 vs. the ctrl group). All data are the representative of three independent experiments

To verify whether NOD1/RIP2 signalling contributed to haemorrhage-associated brain injury, we first investigated the expression of NOD1 and RIP2 in response to ICH. A number of mice at different time points following ICH (1, 3, 5 , and 7 days) were employed for comparison with the sham group. Western blot analysis showed that NOD1 and RIP2 expression was barely detectable within the sham brain, whereas significant overproduction was observed at 1 day following ICH surgery, with a maximum at 3 days and a gradual decrease thereafter (Fig. 2a–b). To further confirm that NOD1 and RIP2 were indeed significantly upregulated in the acute phase of ICH, we explored NOD1 and RIP2 expression in cultured primary microglia and BV2 cells challenged with different doses of hemin (0, 10 μM, 20 μM, 40 μM, 60 μM) for 24 h. Western blot analysis showed that NOD1 and RIP2 levels were both elevated with increasing doses of hemin in primary microglia (Fig. 2c–d) and in BV2 cells (Fig. 2e–f). Therefore, in view of the above results, we speculated that NOD1/RIP2 signalling might participate in the pathological process of ICH.

### NOD1/RIP2 inhibition ameliorated ICH-induced brain damage and microglial activation

**Fig. 3:**
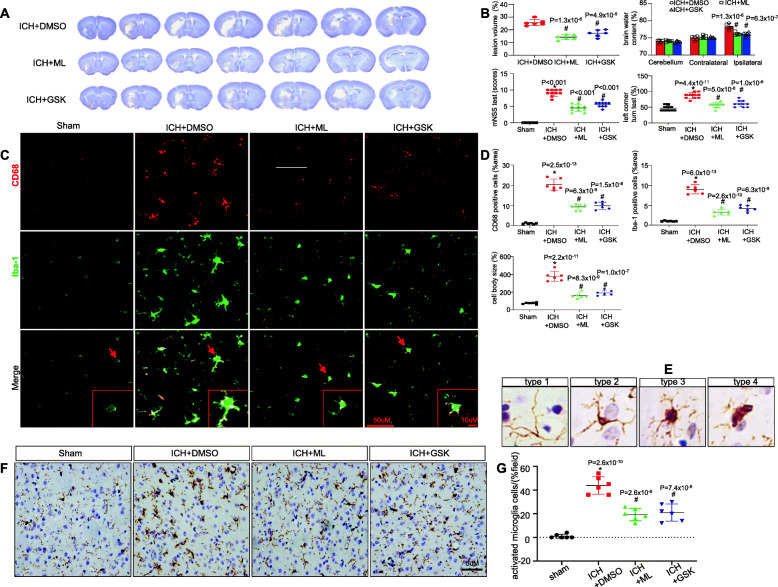
NOD1/RIP2 inhibition alleviated ICH-induced brain damage and microglial activation. **a**, **b** Levels of injury volume, brain water content and neurological function in the indicated group (*n* = 6 mice/group for analysis of injury volume and brain water content; *n* = 10 mice/group for analysis of neurological function. #*P* < 0.01 vs. the DMSO-treated group). **c**, **d** CD68-, Iba-1-positive cells and the cell body size of microglia in the perihematomal tissue by immunofluorescence staining (*n* = 6 mice for each group, 3 images/mouse; *P < 0.01 vs. the sham group, #*P* < 0.01 vs. the DMSO-treated group). **e** The representative images of the four subtypes of microglia: type 1, the resting form of microglia; type 2, the initiated form of activated microglia; type 3, the activated form of microglia with non-phagocytic function; type 4, the overactivated form of microglia with phagocytic function. **f**, **g** Immunohistochemical analysis of microglial subtypes and quantitative data of activated microglia in the indicated groups (*n* = 6 mice for each group, 3 images/mouse; *P < 0.01 vs. the sham group, #*P* < 0.01 vs. the DMSO-treated group). All data are representative of at least six independent experiments

To investigate the role of NOD1/RIP2 signalling in ICH-induced brain damage, ML130 (a specific NOD1 inhibitor) and GSK583 (a specific RIP2 inhibitor) were employed in our study. The optimal concentration of ML130 for inhibiting NOD1 and GSK583 for inhibiting RIP2 was 20 μM, which was based on their effect on the expression of the proinflammatory factor iNOS in BV2 cells in response to hemin (Additional file 1: Figure S1). The impact of NOD1 inhibition and RIP2 inhibition on ICH was first evaluated by the injury volume. There was a significant reduction in injury volume in the ML130- or GSK583-treated group compared with the DMSO-treated group (Fig. 3a, b). In addition, mice subjected to either ML130 or GSK583 administration exhibited a significant reduction in brain water content, neurological deficit scores, and percentage of right turns in the corner-turning experiment compared with the DMSO-treated group (Fig. 3b). Collectively, these results demonstrated that NOD1/RIP2 signalling inhibition might be protective against ICH-induced brain damage. Based on the protective role of NOD1/RIP2 inhibition against ICH-induced brain damage in our study, we further explored the possible molecular mechanism of NOD1/RIP2 involvement. Since the microglia-driven inflammatory response was of great importance in ICH-induced brain damage, we then investigated the role of NOD1/RIP2 signalling in microglial activation after ICH in mice. Immunofluorescence staining for CD68, a marker of activated microglia, together with Iba1, showed that the numbers of cells positive for CD68 and Iba1 were dramatically increased in perihematomal tissue from the ICH group compared with the sham group, whereas were significantly downregulated by either ML130 or GSK583 treatment (Fig. 3c, d). Moreover, the enlarged cell body size of microglia induced by ICH could be reduced following ML130 or GSK583 treatment (Fig. 3c, d). Furthermore, we evaluated microglial morphology by performing Sholl analysis with immunohistochemistry staining. The microglia were defined into four types on the basis of the length and thickness of process [8, 35]. As shown in Fig. 3e, type 1, i.e. the resting form of microglia, the processes are fine and long, and the cell body is small; type 2, i.e. the initiated form of activated microglia, the processes are still long, but not smooth, with many small branches. The cell body size gets bigger; type 3, i.e. the activated form of microglia with non-phagocytic function, the processes become shorter and uneven with various thickness, and the cell body enlarges in bigger and irregular size; type 4, i.e. the overactivated form of microglia with phagocytic function, the processes are thick and short, and the cell body is big and dark. The resting microglia, characterized by small cell bodies and fine and long synapses [8], in the sham group shifted to activated microglia, characterized by significantly enlarged cell bodies and thick, shrunken synapses, in response to ICH [7, 8] (Fig. 3f, g). However, these harmful effects were significantly ameliorated by either ML130 or GSK583 treatment (Fig. 3f, g). Taken together, these results showed that NOD1/RIP2 inhibition alleviated ICH-induced brain damage in part by decreasing microglial activation.

### NOD1 inhibition inhibited the inflammatory factors and JNK/P38 MAPK, NF-κB signalling pathway following ICH

**Fig. 4:**
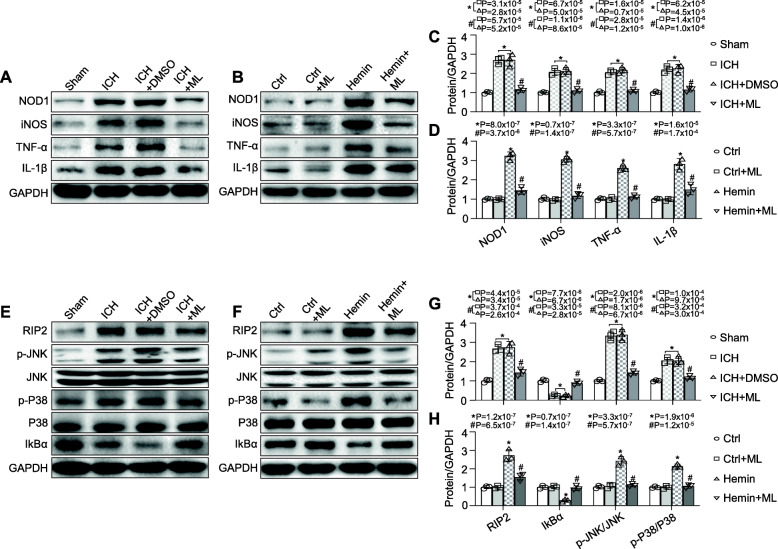
NOD1 inhibition significantly reduced the inflammatory response in response to ICH. Levels of iNOS, TNF-α, and IL-1β protein **a**, **c** in the brain in the indicated groups (*n* = 3 mice for each group; *P < 0.01 vs*.* the sham group, #*P* < 0.01 vs. the ICH/ICH + DMSO group) and **b**, **d** in the primary microglia from the indicated groups (*n* = 3 experiments for each group; *P < 0.01 vs. the ctrl group, #*P* < 0.01 vs. the Hemin group); RIP2, total and phosphorylated JNK/P38 MAPK, and IκBα protein levels **e**, **g** in the brain in the indicated groups (*n* = 3 mice for each group; *P < 0.01 vs. the sham group, #*P* < 0.01 vs*.* the ICH/ICH + DMSO) and **f**, **h** in the primary microglia from the indicated groups (*n* = 3 experiments for each group; *P < 0.01 vs. the ctrl group, #*P* < 0.01 vs. the hemin group). All data are representative of three independent experiments

The ability of NOD1 inhibition to suppress the microglia-driven inflammatory response was further verified by assessing inflammatory factors, including iNOS, IL-1β, and TNF-α. Our study revealed that perihematomal tissue from the non-ML130-treated group induced significantly higher levels of the above inflammatory factors than did those from the sham group. However, these factors were significantly downregulated upon treatment with ML130. There was no significant difference between the ICH + DMSO and ICH groups (Fig. 4a, c). To further validate the results, we also examined the expression of these inflammatory factors in vitro by western blotting. Pre-treatment with ML130 significantly alleviated inflammatory factors that were upregulated in primary microglia in response to hemin, and there was no significant difference between the control and control + ML130 groups (Fig. 4b, d). Since JNK/P38 MAPK and NF-κB signalling were involved in NOD1/RIP2-dependent inflammatory signalling, we further assessed the role of ML130 in these signalling pathways during ICH. Our results showed that JNK/P38 MAPK and NF-κB signalling were activated by ICH. However, in the presence of ML130, significantly lower levels of phosphorylated JNK/P38 MAPK and higher production of IκBα were noted (Fig. 4e, g). A similar outcome was detected in the in vitro study (Fig. 4f, h). These observed changes indicated that NOD1 inhibition protected against ICH-induced brain damage by inhibiting the inflammatory response following ICH.

### RIP2 inhibition alleviated inflammatory factors and JNK/P38 MAPK, NF-κB signalling pathways following ICH

**Fig. 5:**
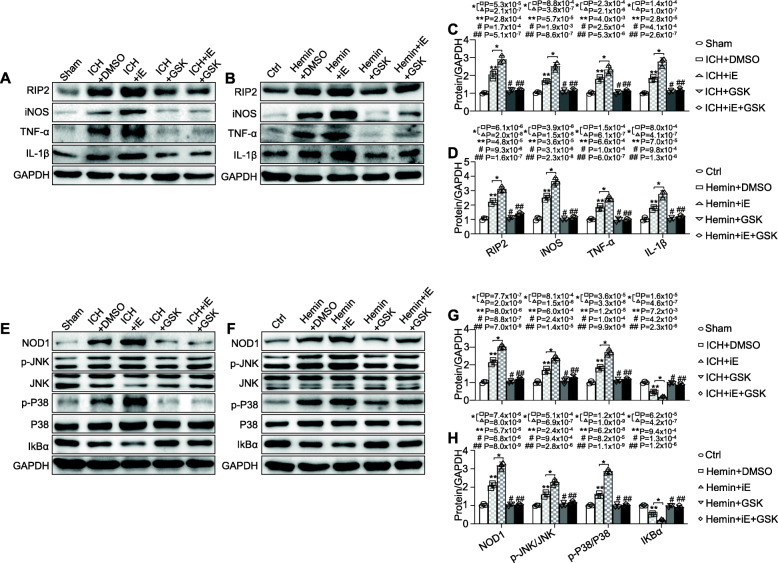
RIP2 inhibition alleviated the inflammatory response induced by NOD1 activation post ICH. Levels of iNOS, TNF-α, and IL-1β protein **a**, **c** in the brain in the indicated groups (*n* = 3 mice for each group; *P < 0.01 vs. the sham group, **P < 0.01 vs. the ICH + iE group, #*P* < 0.01 vs. the ICH + DMSO, ##*P* < 0.01 vs. the Hemin + iE group) and **b**, **d** in cultured primary microglia from the indicated groups (*n* = 3 experiments for each group; *P < 0.01 vs. the ctrl group, **P < 0.01 vs. the Hemin + iE group, #*P* < 0.01 vs. the Hemin + DMSO, ##*P* < 0.01 vs. the Hemin + iE group). Total and phosphorylated JNK/P38 MAPK, IκBα, and NOD1 protein levels **e**, **g** in the brain in the indicated groups (*n* = 3 mice for each group; *P < 0.01 vs. the sham group, **P < 0.01 vs. the ICH + iE group, #*P* < 0.01 vs. the ICH + DMSO, ##*P* < 0.01 vs. the Hemin + iE group) and **f**, **h** in cultured primary microglia from the indicated groups (*n* = 3 experiments for each group; *P < 0.01 vs. the ctrl group, **P < 0.01 vs. the Hemin + iE group, #*P* < 0.01 vs. the Hemin + DMSO, ##*P* < 0.01 vs. the Hemin + iE group). All data are representative of three independent experiments

To address the possibility that NOD1/RIP2 signalling contributed to ICH-induced brain damage, we further defined the role of RIP2, the critical partner of NOD1, in ICH with its inhibitor GSK583. The inflammatory response-associated factors iNOS, IL-1β, and TNF-α were increased in the brain in response to ICH (Fig. 5a, c) and in the primary microglia exposed to hemin (Fig. 5b, d), and this effect was further enhanced by the NOD1 agonist C12-iE-DAP (Fig. 5a–d). However, these harmful effects could be blunted by administration of GSK583 (Fig. 5a–d). In addition, the overactivation of JNK/P38 MAPK and NF-κB activity in the ICH-challenged brain and in the hemin-stimulated primary microglia model was further enhanced by C12-iE-DAP, but simultaneously reversed by treatment with GSK583 (Fig. 5e–h). These results indicated the protective effect of RIP2 inhibition on ICH. However, the upregulation of NOD1 expression post ICH was also significantly reduced after pre-treatment with GSK583 (Fig. 5e, g), although NOD1 was presumably not directly affected by GSK583. The in vitro study in primary microglia yielded similar outcomes (Fig. 5f, h). Since the inflammatory factors IL-1β and TNF-α act as common downstream molecules of NOD1/RIP2 signalling and can be reduced by GSK583, we speculate that the decreased NOD1 expression induced by GSK583 may be attributed to crosstalk between inflammatory factors and NOD1.

### NOD1 and RIP2 expression can be upregulated by IL-1β and TNF-α

**Fig. 6:**
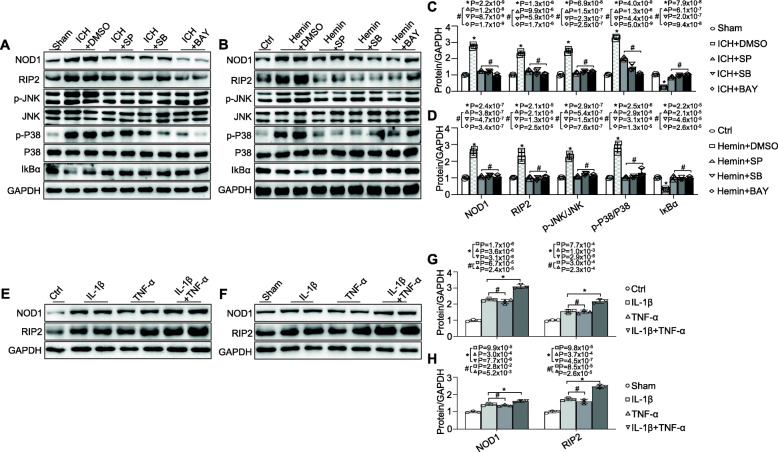
NOD1 and RIP2 expression can be upregulated by IL-1β and TNF-α. Total and phosphorylated JNK/P38 MAPK, IκBα, NOD1, and RIP2 protein levels **a**, **c** in the brain in ICH-induced mice treated with the indicated inhibitors (*n* = 3 mice for each group; *P < 0.01 vs. the sham group, #*P* < 0.01 vs. the ICH + DMSO group) and **b**, **d** in hemin-challenged BV2 cells pretreated with the indicated inhibitors (*n* = 3 experiments for each group; *P < 0.01 vs. the ctrl group, #*P* < 0.01 vs. the Hemin + DMSO group). NOD1 and RIP2 expression **e**, **g** in primary microglia challenged with either TNF-α or IL-1β alone or in combination for 24 h (*n* = 3 experiments for each group; *P < 0.01 vs. the ctrl group, #*P* < 0.01 vs. the IL-1β + TNF-α-challenged group) and **f**, **h** in mice challenged with either TNF-α or IL-1β alone or in combination for 24 h (*n* = 3 mice for each group; *P < 0.01 vs. the sham group, #*P* < 0.01 vs*.* the IL-1β + TNF-α-challenged group). All data are representative of three independent experiments

To further demonstrate whether a crosstalk was present between NOD1/RIP2 signalling and the inflammatory factors, we first applied the inhibitors of JNK kinase (SP600125, 10 μM), P38 kinase (SB202190,10 μM), and NF-κB (BAY11-7082, 10 μM) signalling. These inhibitors were demonstrated to inhibit the production of the inflammatory factors, including iNOS, IL-1β, and TNF-α, in the ICH-challenged brain (Additional file 2: Figure S2A) and in hemin-stimulated BV2 cells (Additional file 2: Figure S2B). Notably, all the three inhibitors could prevent NOD1 and RIP2 upregulation in the brain in response to ICH (Fig. 6a, c) and in BV2 cells in response to hemin (Fig. 6b, d), which was similar to the effects of GSK583. In addition, these kinase inhibitors sufficiently suppressed not only their target but also the other two kinases. For example, pharmacological inhibition of JNK with SP600125 sufficiently suppressed P38 activation and IκBα inactivation in the ICH-challenged brain and in hemin-stimulated BV2 cells, and vice versa (Fig. 6a–d). Overall, we speculate that the reduction in NOD1 and RIP2 expression induced by these three inhibitors (SP600125, SB202190, BAY11-7082) and the reductions in JNK/P38 MAPK and NF-κB activation induced by not only their specific inhibitors but also the other two inhibitors likely resulted from the reduced inflammatory cytokines downstream of NOD1/RIP2 signalling, and that crosstalk might occur between NOD1/RIP2 signalling and inflammatory cytokines. To further explore whether NOD1 and RIP2 were indeed upregulated in the presence of the inflammatory cytokines IL-1β and TNF-α, cultured primary microglia stimulated with IL-1β (20 ng/ml) [36] or TNF-α (20 ng/ml) [37] alone or in combination were investigated, and our results showed significant increases in NOD1 and RIP2 protein levels following 24 h of IL-1β or TNF-α stimulation in primary microglia. A marked synergistic impact on NOD1 and RIP2 expression was detected in response to combined administration of IL-1β plus TNF-α, and there were no significant differences between IL-1β and TNF-α stimulation alone (Fig. 6e, g). The functional interplay between NOD1/RIP2 and inflammatory cytokines was further confirmed in vivo. The mice challenged with IL-1β (50 ng) [38] or TNF-α (50 ng) [39] alone or in combination (local right striatum injection) showed higher NOD1 and RIP2 expression in comparison to the sham group, and the coadministration of IL-1β and TNF-α into mice induced a significant upregulation of NOD1 and RIP2 protein levels compared with the IL-1β- or TNF-α-stimulated group (Fig. 6f, h). Collectively, these results confirmed our above conclusion that NOD1/RIP2 signalling not only induced inflammatory cytokine production but was also affected by inflammatory conditions.

## Discussion

**Fig. 7:**
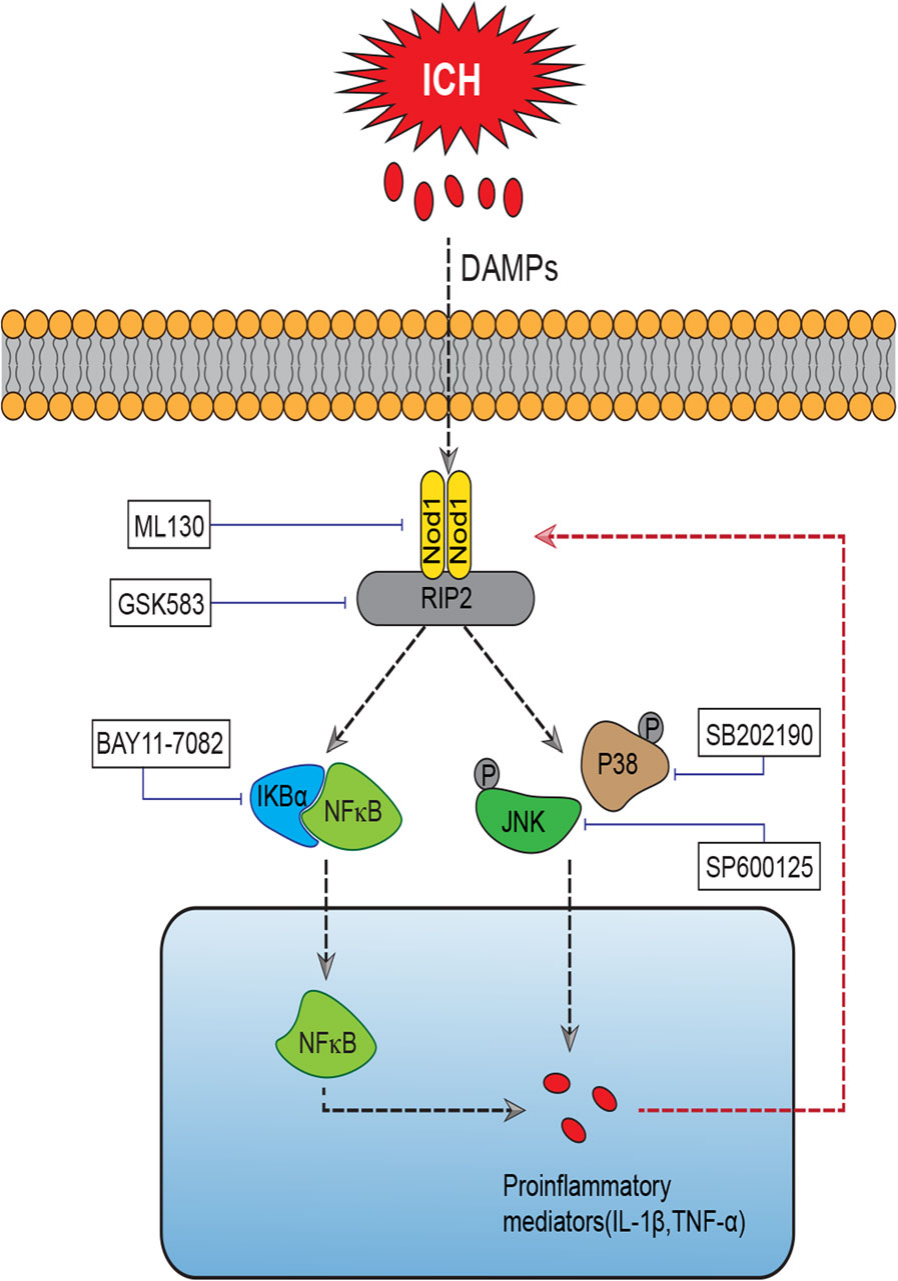
Working model of the molecular mechanisms by which NOD1/RIP2 signalling mediates the ICH-induced inflammatory response and undergoes positive crosstalk with inflammatory factors during ICH in mice. More specifically, ICH-induced damage-associated molecular patterns (DAMPs) were able to initiate the activation of NOD1, which then resulted in the activation of its adaptor RIP2. Activated RIP2 in turn exerted its regulatory effect on the proinflammatory factors TNF-α and IL-1β via JNK/P38 MAPK- and NF-κB-dependent signalling, and the proinflammatory factors TNF-α and IL-1β that were induced by NOD1/RIP2 signalling also enhanced NOD1/RIP2 upregulation. Either the NOD1 inhibitor ML130 or RIP2 inhibitor GSK583 could suppress the inflammatory response induced by ICH. The P38 inhibitor SB202190, JNK inhibitor SP600125, and NF-κB inhibitor BAY11-7082 were used for intervention

Our study provided the first evidence for the role of NOD1/RIP2 signalling in a collagenase-induced ICH model in mice. We found that NOD1 and RIP2 expression was significantly increased in the brains of mice following ICH. Inhibition of either NOD1 or RIP2 significantly protected against ICH-induced brain damage. Such protective effects likely resulted from preventing the transition from the quiescent state of microglia towards the overactivated state and suppressing the production of microglia-associated inflammatory factors, including iNOS, IL-1β, and TNF-α. More significantly, we found for the first time that positive feedback occurred between NOD1/RIP2 and the inflammatory cytokines IL-1β and TNF-α, which coactivated each other and exacerbated the pathogenesis of ICH. Overall, our study showed for the first time that NOD1/RIP2 signalling modulated the microglia-driven proinflammatory response during ICH, and a possible working model for NOD1/RIP2 signalling is shown in Fig. 7.

Increasing evidence has demonstrated that activation of the innate immune system during ICH contributes to neuroinflammation and secondary brain damage [40, 41]. Microglia that act as CNS macrophages are capable of evoking the innate immune response and driving inflammatory mediator production upon sensing danger signals that arise from ICH [7]. NOD1 was originally found to sense bacterial peptidoglycan fragments and result in a robust innate immune response [42]. However, in addition to sensing peptidoglycan, some studies have demonstrated that NOD1 expressed at a low level in stable conditions can be strongly upregulated in liver in response to ischemia [43] and in mesangial cells following exposure to high glucose and LPS [44]. Numerous studies have highlighted the crucial role of NOD1 in renal and liver diseases, in which NOD1 activation can evoke the immune response and amplify tissue damage, while blockade of NOD1 can act as a protective factor against such tissue injury by modulating PMNs [16, 17, 45]. In addition, Chan et al. found that immune cell-specific NOD1 knockout could alleviate adipose tissue inflammation [18]. RIP2, a crucial downstream adaptor of NOD1, is involved in the inflammatory response [46] and acts as an activator of MAPK and NF-κB signalling [47–49]. Some studies have demonstrated that RIP2 blockade can block the production of proinflammatory mediators induced by smoke in the lung [50] and by cardiac hypertrophy [51]. Zhang et al. demonstrated that RIP2 was significantly increased in hypoxia- and ischemia-induced neuronal cells [52]. However, the role of NOD1/RIP2 signalling in the brain, especially when subjected to ICH, is unknown. Our study demonstrated that NOD1/RIP2 signalling participated in the pathological process of ICH and that inhibition of NOD1/RIP2 contributed to the alleviation of ICH-induced brain damage. This view was based on the following observations. First, NOD1 and RIP2 expression exhibited a marked increase in the brains of ICH-challenged mice and in hemin-stimulated cultured primary microglia. This increase was accompanied by enhanced microglial activation and proinflammatory factor production. Second, either inhibition of NOD1 by its inhibitor ML130 or inhibition of RIP2 by its inhibitor GSK583 caused reduced microglial activation, a smaller injury volume, decreased brain water content, and improved neurologic function compared with the DMSO-treated group. Third, either the NOD1 inhibitor or RIP2 inhibitor exerted an anti-inflammatory effect (reduced IL-1β and TNF-α production) on ICH, while the NOD1 agonist iE-DAP exerted the opposite effect. Fourth, JNK/P38 MAPK and NF-κB, which are activated and play proinflammatory roles in the progression of ICH [53, 54], can be suppressed upon administration of ML130 or GSK583. These findings supported the important role of NOD1/RIP2 in modulating JNK/P38 MAPK and NF-κB activity. Therefore, we assumed that NOD1/RIP2 signalling might play an important role in the secondary inflammatory response during ICH.

However, and more importantly, we demonstrated that in addition to modulating TNF-α/IL-1β production, NOD1 and RIP2 were also targets of regulation by TNF-α/IL-1β. A positive feedback loop was observed between NOD1/RIP2 and TNF-α/IL-1β. This conclusion was based on the following results. First, we found that the RIP2 inhibitor GSK583 not only inhibited RIP2 expression, but also significantly suppressed NOD1 upregulation in vivo and in vitro in an ICH model, which was a theoretical contradiction against the previous finding that NOD1, a proven upstream factor of RIP2, should not be affected by a RIP2 inhibitor. Second, a similar effect was demonstrated in the presence of JNK/P38 MAPK kinases and NF-κB signalling inhibitors. We found that the inhibitors of JNK/P38 MAPK and NF-κB not only inhibited their targets but also the two other factors. In addition, these inhibitors also negatively regulated the expression of NOD1 and RIP2. Third, our study demonstrated that administration of either TNF-α or IL-1β in mice and cultured primary microglia could upregulate NOD1 and RIP2 expression. Consistent with our findings, previous studies found that treatment of intestinal epithelial cells with TNF-α or treatment of a series of immune cells with IL-1β led to a significant increase in expression of NOD2 (another member of the NLR family) [36, 55]. The possible mechanism responsible for NOD1 and RIP2 upregulation by TNF-α/IL-1β may be as follows. First, the interaction between TNF-α and the TNF-α receptor or between IL-1β and the IL-1 receptor can trigger the activation of NF-κB [36, 55], which contributes to the upregulation of NOD1 and its adaptor RIP2. Second, endoplasmic reticulum (ER) stress, which can be initiated in response to TNF-α/IL-1β [56, 57], was recently reported to contribute to the interaction of tumour necrosis factor (TNF) receptor-associated factor 2 (TRAF2) with NOD1 and lead to NOD1 activation [58, 59]. However, the underlying mechanism by which TNF-α/IL-1β induced NOD1 and RIP2 upregulation at the molecular level remains to be further explored. Overall, the positive feedback loop between NOD1/RIP2 and TNF-α/IL-1β could explain why RIP2 inhibitor resulted in the suppression of NOD1 and JNK/P38 MAPK and NF-κB inhibitors resulted in the suppression of NOD1 and RIP2. However, a “chicken or the egg” issue exists, because the activation of NOD1/RIP2 in response to ICH could be a consequence or a cause of the inflammatory response. There is still insufficient evidence to demonstrate whether NOD1 and RIP2 upregulation occurred before TNF-α/IL-1β production or to elucidate how NOD1 and RIP2 were activated in response to ICH. The answer to the question requires further investigation.

There are a few limitations of our study. First, the inhibitors applied in our study may have off-function targets, and thus, genetic knockout of the target gene will be a better approach to validate our outcome. Second, in the in vitro experiments, we employed hemin to establish the in vitro ICH model to demonstrate the role of NOD1/RIP2 signalling. However, other hematoma components, such as thrombin and haemoglobin, were not applied in our study, and it will be important to employ them in future studies to gain comprehensive insight into the role of NOD1/RIP2 signalling during ICH.

## Conclusions

Our results provide the first evidence that NOD1/RIP2 inhibition is protective against ICH-induced brain injury. This protective effect may result from suppression of the microglia-induced inflammatory response. Furthermore, crosstalk occurred between NOD1/RIP2 and TNF-α/IL-1β during ICH, which was to some extent responsible for the sustained inflammation during ICH and revealed that NOD1/RIP2 signalling represented an important potential therapeutic target for combatting ICH-associated neuroinflammation.

## Supplementary Information

**Additional file 1:** **Figure S1.** The optimal concentration of the NOD1 inhibitor ML130 and the RIP2 inhibitor GSK583 for suppressing the inflammatory response was 20 μM. **(A)** In hemin-induced BV2 cells, ML130 concentrations of 0, 10, 20, and 30 μM were employed to assess the optimal dose of ML130 in inhibiting NOD1 and iNOS expression (n=3 experiments for each group; *P<0.01 vs. the ctrl group, #*P*<0.01 vs*.* the Hemin+ML(0 μM) group). **(B)** In hemin-induced BV2 cells, GSK583 at concentrations of 0, 10, 20, and 30 μM was used to assess the optimal dose of GSK583 in inhibiting RIP2 and iNOS expression (*n*=3 experiments for each group; *P<0.01 vs. the ctrl group, #*P*<0.01 vs*.* the Hemin+GSK(0 μM)). All Data are representative of three independent experiments.

**Additional file 2:** **Figure S2.** Inhibitors of JNK/P38 kinases and NF-κB sufficiently suppressed inflammatory factors following ICH. Levels of iNOS, TNF-α, and IL-1β protein **(A)** in the brain in ICH-induced mice that were treated with the indicated inhibitors (*n*=3 mice for each group; *P<0.01 vs. the sham group, #*P*<0.01 vs*.* the ICH+DMSO group), and **(B)** in hemin-challenged BV2 cells pretreated with the indicated inhibitors (*n*=3 experiments for each group; *P<0.01 vs. the ctrl group, #*P*<0.01 vs. the Hemin+DMSO group). All Data are representative of three independent experiments.

## Data Availability

The datasets used and/or analysed during the present study are available from the corresponding author upon reasonable request.

## Abbreviations

ICH: Intracerebral haemorrhage
NOD1: Dendritic cell-associated C-type lectin-1
RIP2: Receptor-interacting protein 2
IL-1β: Interleukin-1 beta
TNF-α: Tumour necrosis factor-α
iNOS: Inducible nitric oxide synthase
NF-κB: Nuclear factor kappa B
MAPK: Mitogen-activated protein kinase
p-P38: Phosphorylated P38
JNK: c-Jun N-terminal kinases
p-JNK: Phosphorylated JNK
IκBα: Inhibitor of κBα
DAMPs: Damage-associated molecular patterns
DMSO: Dimethyl sulfoxide
mNSS: Modified neurological severity score
PBS: Phosphate-buffered saline
GAPDH: Glyceraldehyde 3-phosphate dehydrogenase

## References

[1] van Asch CJ, Luitse MJ (2010). al. e: Incidence, case fatality, and functional outcome of intracerebral haemorrhage over time, according to age, sex, and ethnic origin: a systematic review and meta-analysis. Lancet Neurol.

[2] Wang X, Arima H, Yang J, Zhang S, Wu G, Woodward M, Munoz-Venturelli P, Lavados PM, Stapf C, Robinson T (2015). Mannitol and outcome in intracerebral hemorrhage: propensity score and multivariable intensive blood pressure reduction in acute cerebral hemorrhage trial 2 results. Stroke..

[3] Bhatia HS, Baron J, Hagl S, Eckert GP, Fiebich BL (2016). Rice bran derivatives alleviate microglia activation: possible involvement of MAPK pathway. J Neuroinflammation.

[4] Xiong XY, Liu L, Yang QW (2016). Functions and mechanisms of microglia/macrophages in neuroinflammation and neurogenesis after stroke. Prog Neurobiol.

[5] Wang J (2010). Preclinical and clinical research on inflammation after intracerebral hemorrhage. Prog Neurobiol.

[6] van Rossum D, Hanisch UK (2004). Microglia. Metab Brain Dis.

[7] Parada E, Casas AI, Palomino-Antolin A, Gómez-Rangel V, Rubio-Navarro A, Farré-Alins V, Narros-Fernandez P, Guerrero-Hue M, Moreno JA, Rosa JM (2019). Early toll-like receptor 4 blockade reduces ROS and inflammation triggered by microglial pro-inflammatory phenotype in rodent and human brain ischaemia models. Br J Pharmacol.

[8] Xu XE, Liu L, Wang YC, Wang CT, Zheng Q, Liu QX, Li ZF, Bai XJ, Liu XH (2019). Caspase-1 inhibitor exerts brain-protective effects against sepsis-associated encephalopathy and cognitive impairments in a mouse model of sepsis. Brain Behav Immun.

[9] Scott G, Zetterberg H, Jolly A, Cole JH, De Simoni S, Jenkins PO, Feeney C, Owen DR, Lingford-Hughes A, Howes O (2018). Minocycline reduces chronic microglial activation after brain trauma but increases neurodegeneration. Brain..

[10] Varnum MM, Ikezu T (2012). The classification of microglial activation phenotypes on neurodegeneration and regeneration in Alzheimer’s disease brain. Arch Immunol Ther Exp (Warsz).

[11] Ponomarev ED, Veremeyko T, Weiner HL (2013). MicroRNAs are universal regulators of differentiation, activation, and polarization of microglia and macrophages in normal and diseased CNS. Glia..

[12] Goerdt S, Politz O, Schledzewski K, Birk R, Gratchev A, Guillot P, Hakiy N, Klemke CD, Dippel E, Kodelja V, Orfanos CE (1999). Alternative versus classical activation of macrophages. Pathobiology..

[13] Chamaillard M, Hashimoto M, Horie Y, Masumoto J, Qiu S, Saab L, Ogura Y, Kawasaki A, Fukase K, Kusumoto S (2003). An essential role for NOD1 in host recognition of bacterial peptidoglycan containing diaminopimelic acid. Nat Immunol.

[14] Carneiro LA, Magalhaes JG, Tattoli I, Philpott DJ, Travassos LH (2008). Nod-like proteins in inflammation and disease. J Pathol.

[15] Moreno L, McMaster SK, Gatheral T, Bailey LK, Harrington LS, Cartwright N, Armstrong PC, Warner TD, Paul-Clark M, Mitchell JA (2010). Nucleotide oligomerization domain 1 is a dominant pathway for NOS2 induction in vascular smooth muscle cells: comparison with Toll-like receptor 4 responses in macrophages. Br J Pharmacol.

[16] Lassailly G, Bou Saleh M, Leleu-Chavain N, Ningarhari M, Gantier E, Carpentier R, Artru F, Gnemmi V, Bertin B, Maboudou P (2019). Nucleotide-binding oligomerization domain 1 (NOD1) modulates liver ischemia reperfusion through the expression adhesion molecules. J Hepatol.

[17] Dharancy S, Body-Malapel M, Louvet A, Berrebi D, Gantier E, Gosset P, Viala J, Hollebecque A, Moreno C, Philpott DJ (2010). Neutrophil migration during liver injury is under nucleotide-binding oligomerization domain 1 control. Gastroenterology.

[18] Chan KL, Tam TH, Boroumand P, Prescott D, Costford SR, Escalante NK, Fine N, Tu Y, Robertson SJ, Prabaharan D (2017). Circulating NOD1 activators and hematopoietic NOD1 contribute to metabolic inflammation and insulin resistance. Cell Rep.

[19] Strober W, Murray PJ, Kitani A, Watanabe T (2006). Signalling pathways and molecular interactions of NOD1 and NOD2. Nat Rev Immunol.

[20] Park JH, Kim YG, McDonald C, Kanneganti TD, Hasegawa M, Body-Malapel M, Inohara N, Nunez G (2007). RICK/RIP2 mediates innate immune responses induced through Nod1 and Nod2 but not TLRs. J Immunol.

[21] Le Bourhis L, Benko S, Girardin SE (2007). Nod1 and Nod2 in innate immunity and human inflammatory disorders. Biochem Soc Trans.

[22] Philpott DJ, Sorbara MT, Robertson SJ, Croitoru K, Girardin SE (2014). NOD proteins: regulators of inflammation in health and disease. Nat Rev Immunol.

[23] Harikrishnan H, Jantan I, Haque MA, Kumolosasi E (2018). Anti-inflammatory effects of hypophyllanthin and niranthin through downregulation of NF-κB/MAPKs/PI3K-Akt signaling pathways. Inflammation..

[24] Zhong L, Zhang ZL, Li X, Liao C, Mou P, Wang T, Wang Z, Wang Z, Wei M, Xu H (2017). TREM2/DAP12 complex regulates inflammatory responses in microglia via the JNK signaling pathway. Front Aging Neurosci.

[25] Turner NA, Blythe NM. Cardiac Fibroblast p38 MAPK: A critical regulator of myocardial remodeling. J Cardiovasc Dev Dis. 2019;6. 10.3390/jcdd6030027 PMC6787752 31394846

[26] Wu CH, Shyue SK, Hung TH, Wen S, Lin CC, Chang CF, Chen SF (2017). Genetic deletion or pharmacological inhibition of soluble epoxide hydrolase reduces brain damage and attenuates neuroinflammation after intracerebral hemorrhage. J Neuroinflammation.

[27] Wan S, Cheng Y, Jin H, Guo D, Hua Y, Keep RF, Xi G (2016). Microglia activation and polarization after intracerebral hemorrhage in mice: the role of protease-activated receptor-1. Transl Stroke Res.

[28] Chu X, Wu X, Feng H, Zhao H, Tan Y, Wang L, Ran H, Yi L, Peng Y, Tong H (2018). Coupling between interleukin-1R1 and necrosome complex involves in hemin-induced neuronal necroptosis after intracranial hemorrhage. Stroke..

[29] Karuppagounder SS, Alin L, Chen Y, Brand D, Bourassa MW, Dietrich K, Wilkinson CM, Nadeau CA, Kumar A, Perry S (2018). N-acetylcysteine targets 5 lipoxygenase-derived, toxic lipids and can synergize with prostaglandin E(2) to inhibit ferroptosis and improve outcomes following hemorrhagic stroke in mice. Ann Neurol.

[30] He Y, Xu L, Li B, Guo ZN, Hu Q, Guo Z, Tang J, Chen Y, Zhang Y, Tang J, Zhang JH (2015). Macrophage-inducible C-type lectin/spleen tyrosine kinase signaling pathway contributes to neuroinflammation after subarachnoid hemorrhage in rats. Stroke..

[31] Li Q, Han X, Lan X, Gao Y, Wan J, Durham F, Cheng T, Yang J, Wang Z, Jiang C (2017). Inhibition of neuronal ferroptosis protects hemorrhagic brain. JCI Insight.

[32] Yang H, Ni W, Jiang H, Lei Y, Su J, Gu Y, Zhou L (2018). Histone deacetylase inhibitor scriptaid alleviated neurological dysfunction after experimental intracerebral hemorrhage in mice. Behav Neurol.

[33] Lan X, Han X, Li Q, Li Q, Gao Y, Cheng T, Wan J, Zhu W, Wang J (2017). Pinocembrin protects hemorrhagic brain primarily by inhibiting toll-like receptor 4 and reducing M1 phenotype microglia. Brain Behav Immun.

[34] Ye XC, Hu JX, Li L, Li Q, Tang FL, Lin S, Sun D, Sun XD, Cui GY, Mei L, Xiong WC (2018). Astrocytic Lrp4 (low-density lipoprotein receptor-related protein 4) contributes to ischemia-induced brain injury by regulating ATP release and adenosine-A2AR (adenosine A2A receptor) signaling. Stroke..

[35] Sanchez-Guajardo V, Febbraro F, Kirik D, Romero-Ramos M (2010). Microglia acquire distinct activation profiles depending on the degree of alpha-synuclein neuropathology in a rAAV based model of Parkinson’s disease. PLoS One.

[36] Li S, Deng P, Wang M, Liu X, Jiang M, Jiang B, Yang L, Hu J (2019). IL-1alpha and IL-1beta promote NOD2-induced immune responses by enhancing MAPK signaling. Lab Investig.

[37] Chen X, Xiao Z, Xie X, Liu X, Jiang M, Yuan C, Yang L, Hu J (2018). TNF-α-induced NOD2 and RIP2 contribute to the up-regulation of cytokines induced by MDP in monocytic THP-1 Cells. J Cell Biochem.

[38] Biondo C, Mancuso G, Midiri A, Signorino G, Domina M, Lanza Cariccio V, Mohammadi N, Venza M, Venza I, Teti G, Beninati C (2014). The interleukin-1beta/CXCL1/2/neutrophil axis mediates host protection against group B streptococcal infection. Infect Immun.

[39] Constantin CE, Mair N, Sailer CA, Andratsch M, Xu ZZ, Blumer MJ, Scherbakov N, Davis JB, Bluethmann H, Ji RR, Kress M (2008). Endogenous tumor necrosis factor alpha (TNFalpha) requires TNF receptor type 2 to generate heat hyperalgesia in a mouse cancer model. J Neurosci.

[40] Giordano KR, Denman CR, Dollish HK, Fernandez F, Lifshitz J, Akhter M, Rowe RK (2020). Intracerebral hemorrhage in the mouse altered sleep-wake patterns and activated microglia. Exp Neurol.

[41] Sansing LH, Harris TH, Welsh FA, Kasner SE, Hunter CA, Kariko K (2011). Toll-like receptor 4 contributes to poor outcome after intracerebral hemorrhage. Ann Neurol.

[42] Caruso R, Warner N, Inohara N, Núñez G (2014). NOD1 and NOD2: signaling, host defense, and inflammatory disease. Immunity..

[43] Xi J, Yan M, Li S, Song H, Liu L, Shen Z, Cai JZ (2019). NOD1 activates autophagy to aggravate hepatic ischemia-reperfusion injury in mice. J Cell Biochem.

[44] Huang W, Gou F, Long Y, Li Y, Feng H, Zhang Q, Gao C, Chen G, Xu Y (2016). High glucose and lipopolysaccharide activate NOD1-RICK-NF-κB inflammatory signaling in mesangial cells. Exp Clin Endocrinol Diabetes.

[45] Shigeoka AA, Kambo A, Mathison JC, King AJ, Hall WF, da Silva CJ, Ulevitch RJ, McKay DB (2010). Nod1 and nod2 are expressed in human and murine renal tubular epithelial cells and participate in renal ischemia reperfusion injury. J Immunol.

[46] Shimada K, Chen S, Dempsey PW, Sorrentino R, Alsabeh R, Slepenkin AV, Peterson E, Doherty TM, Underhill D, Crother TR, Arditi M (2009). The NOD/RIP2 pathway is essential for host defenses against *Chlamydophila pneumoniae* lung infection. PLoS Pathog.

[47] Usluoglu N, Pavlovic J, Moelling K, Radziwill G (2007). RIP2 mediates LPS-induced p38 and IkappaBalpha signaling including IL-12 p40 expression in human monocyte-derived dendritic cells. Eur J Immunol.

[48] Lu C, Wang A, Dorsch M, Tian J, Nagashima K, Coyle AJ, Jaffee B, Ocain TD, Xu Y (2005). Participation of Rip2 in lipopolysaccharide signaling is independent of its kinase activity. J Biol Chem.

[49] Krieg A, Correa RG, Garrison JB, Le Negrate G, Welsh K, Huang Z, Knoefel WT, Reed JC (2009). XIAP mediates NOD signaling via interaction with RIP2. Proc Natl Acad Sci U S A.

[50] Dong J, Liao W, Tan LH, Yong A, Peh WY, Wong WSF (2019). Gene silencing of receptor-interacting protein 2 protects against cigarette smoke-induced acute lung injury. Pharmacol Res.

[51] Zhao CH, Ma X, Guo HY, Li P, Liu HY (2017). RIP2 deficiency attenuates cardiac hypertrophy, inflammation and fibrosis in pressure overload induced mice. Biochem Biophys Res Commun.

[52] Zhang WH, Wang X, Narayanan M, Zhang Y, Huo C, Reed JC, Friedlander RM (2003). Fundamental role of the Rip2/caspase-1 pathway in hypoxia and ischemia-induced neuronal cell death. Proc Natl Acad Sci U S A.

[53] Chen S, Zhao L, Sherchan P, Ding Y, Yu J, Nowrangi D, Tang J, Xia Y, Zhang JH (2018). Activation of melanocortin receptor 4 with RO27-3225 attenuates neuroinflammation through AMPK/JNK/p38 MAPK pathway after intracerebral hemorrhage in mice. J Neuroinflammation.

[54] Yin M, Chen Z, Ouyang Y, Zhang H, Wan Z, Wang H, Wu W, Yin X (2017). Thrombin-induced, TNFR-dependent miR-181c downregulation promotes MLL1 and NF-kappaB target gene expression in human microglia. J Neuroinflammation.

[55] Rosenstiel P, Fantini M, Brautigam K, Kuhbacher T, Waetzig GH, Seegert D, Schreiber S (2003). TNF-alpha and IFN-gamma regulate the expression of the NOD2 (CARD15) gene in human intestinal epithelial cells. Gastroenterology..

[56] Eizirik DL, Cardozo AK, Cnop M (2008). The role for endoplasmic reticulum stress in diabetes mellitus. Endocr Rev.

[57] Chae MK, Park SG, Song SO, Kang ES, Cha BS, Lee HC, Lee BW (2012). Pentoxifylline attenuates methionine- and choline-deficient-diet-induced steatohepatitis by suppressing TNF-alpha expression and endoplasmic reticulum stress. Exp Diabetes Res.

[58] Caruso R, Nunez G (2016). Innate Immunity: ER stress recruits NOD1 and NOD2 for delivery of inflammation. Curr Biol.

[59] Mendez JM, Kolora LD, Lemon JS, Dupree SL, Keestra-Gounder AM. Activation of the endoplasmic reticulum stress response impacts the NOD1 signaling pathway. Infect Immun. 2019;87. 10.1128/IAI.00826-18 PMC6652781 31109951

