# Supplementary figures and images for: NOD1/RIP2 signalling enhances the microglia-driven inflammatory response and undergoes crosstalk with inflammatory cytokines to exacerbate brain damage following intracerebral haemorrhage in mice
Source: J Neuroinflammation. 2020 Dec 1;17:364. doi: 10.1186/s12974-020-02015-9 (PMC7708246; doi:10.1186/s12974-020-02015-9)

**A**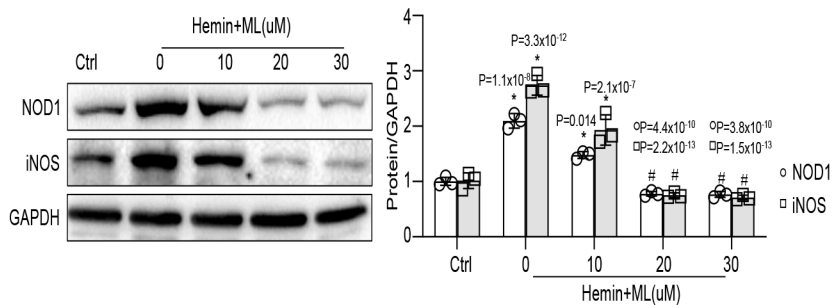**B**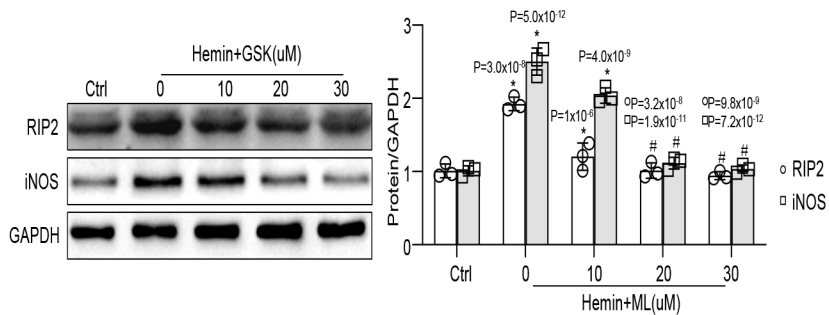

Supplement: Supplementary file 1 — Additional file 1: Figure S1. The optimal concentration of the NOD1 inhibitor ML130 and the RIP2 inhibitor GSK583 for suppressing the inflammatory response was 20 μM. (A) In hemin-induced BV2 cells, ML130 concentrations of 0, 10, 20, and 30 μM were employed to assess the optimal dose of ML130 in inhibiting NOD1 and iNOS expression (n=3 experiments for each group; *P<0.01 vs. the ctrl group, #P<0.01 vs. the Hemin+ML(0 μM) group). (B) In hemin-induced BV2 cells, GSK583 at concentrations of 0, 10, 20, and 30 μM was used to assess the optimal dose of GSK583 in inhibiting RIP2 and iNOS expression (n=3 experiments for each group; *P<0.01 vs. the ctrl group, #P<0.01 vs. the Hemin+GSK(0 μM)). All Data are representative of three independent experiments. [file 12974_2020_2015_MOESM1_ESM.pdf]

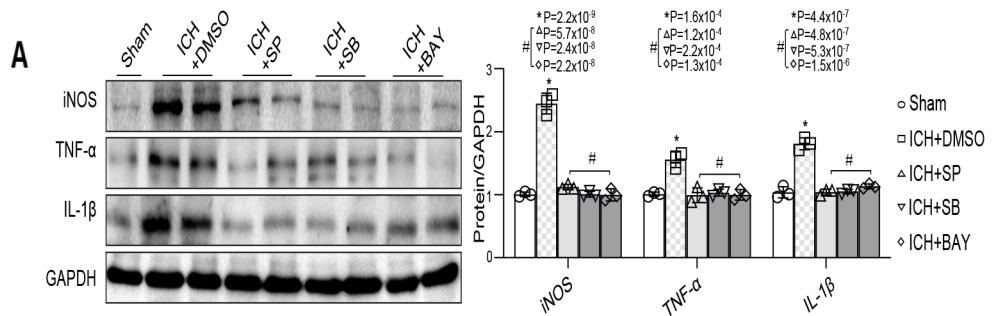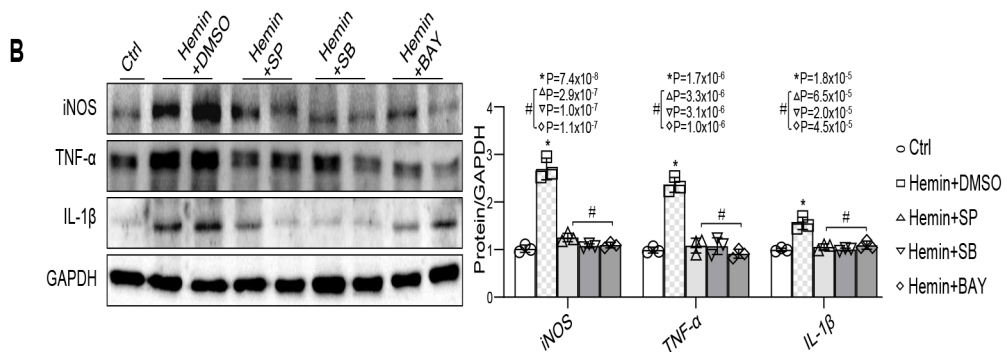

Supplement: Supplementary file 2 — Additional file 2: Figure S2. Inhibitors of JNK/P38 kinases and NF-κB sufficiently suppressed inflammatory factors following ICH. Levels of iNOS, TNF-α, and IL-1β protein (A) in the brain in ICH-induced mice that were treated with the indicated inhibitors (n=3 mice for each group; *P<0.01 vs. the sham group, #P<0.01 vs. the ICH+DMSO group), and (B) in hemin-challenged BV2 cells pretreated with the indicated inhibitors (n=3 experiments for each group; *P<0.01 vs. the ctrl group, #P<0.01 vs. the Hemin+DMSO group). All Data are representative of three independent experiments. [file 12974_2020_2015_MOESM2_ESM.pdf]
